# A novel digital workflow to fabricate anterior repositioning splint with fully masticatory function based on virtual dental patient for anterior disc displacement with reduction: a case report

**DOI:** 10.3389/froh.2025.1534571

**Published:** 2025-03-12

**Authors:** Weicai Liu, Xinhuan Zhao, Jiefei Shen, Ran Wei

**Affiliations:** ^1^ Shanghai Engineering Research Center of Tooth Restoration and Regeneration & Tongji Research Institute of Stomatology & Department of Prosthodontics, Shanghai Tongji Stomatological Hospital and Dental School, Tongji University, Shanghai, China; ^2^ State Key Laboratory of Oral Diseases, National Clinical Research Center for Oral Diseases, Department of Prosthodontics, West China Hospital of Stomatology, Sichuan University, Chengdu, Sichuan, China

**Keywords:** temporomandibular disorders, anterior disc displacement with reduction, functional anterior repositioning splint, virtual dental patient, case report

## Abstract

**Introduction:**

Temporomandibular disorders (TMDs) refer to a range of conditions caused by anatomical and functional abnormalities of the temporomandibular joints. Anterior disc displacement with reduction (ADDwR) is one of the most common TMDs, characterized by symptoms such as jaw pain and audible clicking during jaw movements.

**Case report:**

A 38-year-old female was admitted to our hospital in April 2023 with a chief complaint of joint snapping and jaw pain persisting for nearly a decade. After a comprehensive examination, she was diagnosed with ADDwR of bilateral temporomandibular joints. The patient then underwent functional anterior repositioning splint (FARS) therapy, which continued until July 2023, resulting in complete symptom remission. She is currently undergoing additional orthodontic treatment.

**Conclusion:**

Anterior repositioning splint (ARS) therapy used to be considered one of the most effective therapies for ADDwR. However, concerns have emerged regarding its dependence on clinicians' subjective experience, potential inaccuracies in ARS design, and impact on patients' masticatory function. This case has improved the traditional ARS therapy with the establishment of a novel digital workflow to fabricate ARS with a fully masticatory function based on the virtual dental patient (VDP) for ADDwR. The FARS workflow ensured predictability and visualization of treatment effect through digital simulation, enhanced the personalization and precision of FARS design via VDP, and preserved the patient's masticatory function through the tailored design of occlusal surface morphology.

## 1 Introduction

Temporomandibular disorders (TMDs) refer to a prevalent category of conditions affecting the oral and maxillofacial regions, characterized by a variety of etiologies and implications for the physical and psychological well-being of patients (1). Anterior disc displacement with reduction (ADDwR) is a type of TMDs wherein the temporomandibular joint disc, situated between the mandible and the skull, is misaligned (2, 3), and it accounts for a significant portion of diagnoses (4). The disc of the ADDwR patient moves out of its normal position when the jaw is opened and returns to its normal position when closed, resulting in a clicking sound, pain, or discomfort (5). The diagnosis relies on both clinical and medical imaging examinations to clarify the relationship between the disc and condyle.

Anterior repositioning splint (ARS) therapy is a commonly used conservative treatment for ADDwR, aimed at realigning the condyle and the anterior displaced disc to form a new stable disc-condyle relationship, thereby reducing the disc to the right position (6). However, recent research have pointed out that the application of ARS therapy is based solely on the subjective experiences of clinicians, with a notable absence of standardized protocols (7). Moreover, the lack of established guidelines for treatment procedures and follow-up care often presents challenges for both patients and clinicians (8). Herein, in this context, we presented a case report of a 38-year-old female diagnosed with ADDwR, who achieved resolution of her condition through the novel FARS therapy. This therapy involved a comprehensive assessment of the temporomandibular joint (TMJ) structure and function in patients prior to treatment and entailed the development of individualized treatment protocols and the innovative application of the virtual dental patient (VDP) model. This model integrated intraoral scanning, imaging, and a system for recording and analyzing mandibular movements to accurately determine the positional relationships among the condyle, disc, and articular fossa, as well as to capture the actual trajectories of mandibular movement. The VDP model enabled the visualization and modification of the patient's maxilla and mandible, assisting in the design and fabrication of FARS, ensuring effective chewing functionality daily. This unique case not only provides insights into the improvement of the conventional ARS therapies but also emphasizes the advantages for integrating digital technology into treatment modalities.

## 2 Case report

The patient, a 38-year-old woman, had a chief complaint of joint snapping and jaw pain. She had endured for nearly ten years the pain in front of the ears on both sides occasionally, and she was under great stress. The jaw pain was greater during the menstrual period. The patient did not accept any interventions before (Supplementary Figure S1).

None of the information that reveals the patient's identity was disclosed. The participant had given her consent for a thorough evaluation and publication of the report as well.

### 2.1 Initial clinical examination and diagnosis

This patient was diagnosed with anterior disc displacement with reception of bilateral TMJ, according to the MRI of the patient which showed that the positions of the bilateral discs were inward and there was no obvious abnormal signal in the bone of the bilateral condyles (Supplementary Figure S2).

### 2.2 Therapeutic intervention

Following a comprehensive assessment of the patient's condition, we provided a detailed explanation of the ADDwR treatment. The patient agreed to proceed with the FARS therapy, with understanding of the associated time commitment, financial implications, potential risks, and expected outcomes.

#### 2.2.1 Establishment of VDP

The patient's VDP model was established digital design software (Exocad, Exocad GmbH, Germany), using data obtained from digital dental models, three-dimensional (3D) jawbone models, and recordings of mandibular movements. Subsequent treatment guidance was provided after we intuitively observed and analyzed the patient's temporomandibular joints.

The digital dental model, including the subject's upper and lower jaw dentition, was recorded by an intraoral scanner (3shape, TRIOS 5, Denmark). A 3D jawbone model was constructed from CBCT images by digital segmentation software (InVesalius3.1, Brasilia). And the mandibular movements were recorded by a dynamic occlusal record analysis software (Modjaw, Tech in Motion, Franch).

**Figure 1:**
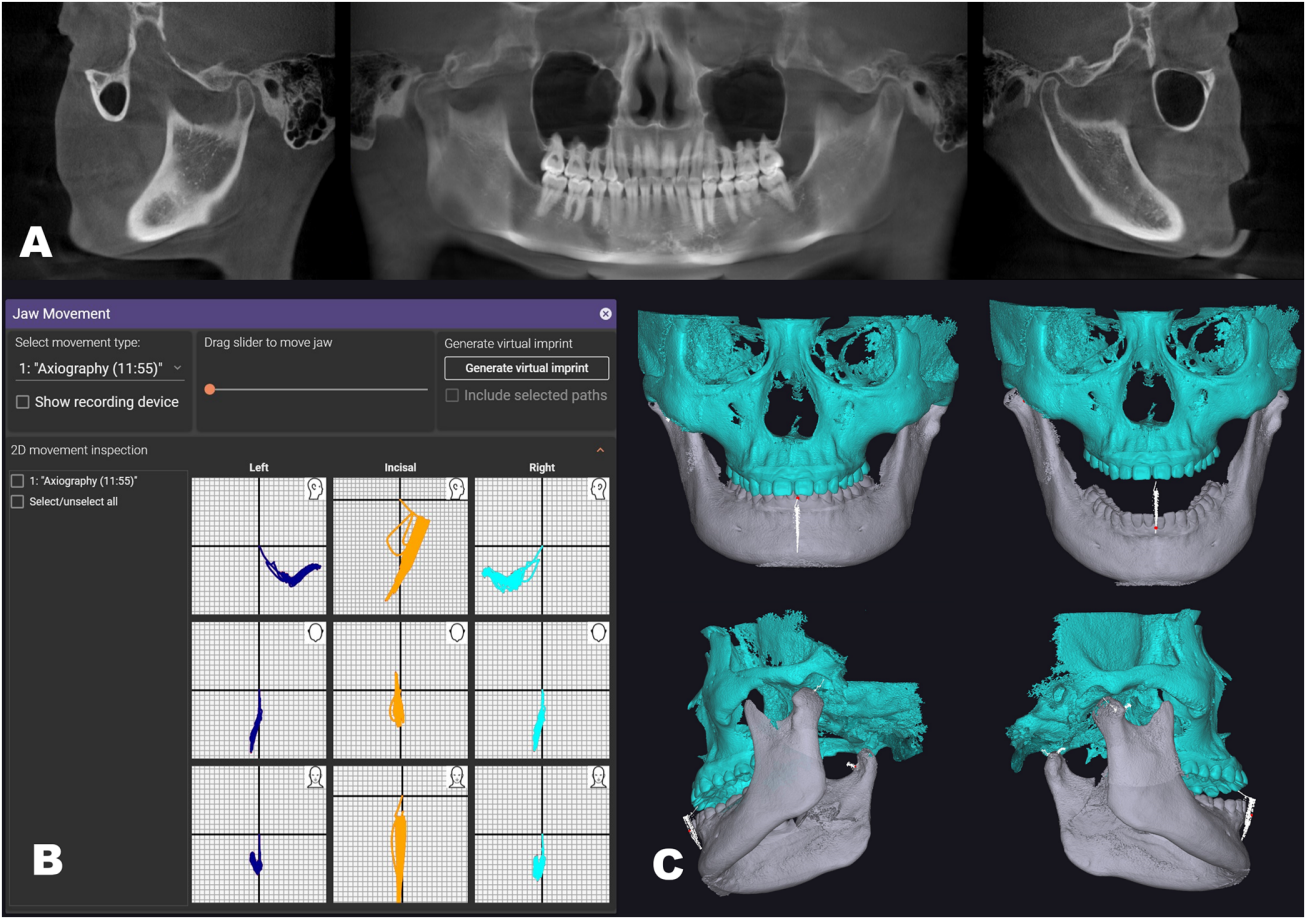
The VDP was developed through the integration of multimodal data fusion. **(A)** CBCT of the patient for diagnostic purposes and model creation. **(B)** Recordings of mandibular movement, timeline for tracking condyle location; **(C)** the VDP model had been established, allowing the mandible to move along recordings of mandibular movement (indicated by the white line).

With the intervention of mandibular movement records, we were able to analyze the variations in both dynamic and static occlusal contact points between the upper and lower dental arches from multiple perspectives throughout the movement process and analyze the occlusal interference that occurs during non-working or forward extension movements (Figure 1).

#### 2.2.2 Design and fabrication of FARS

**Figure 2:**
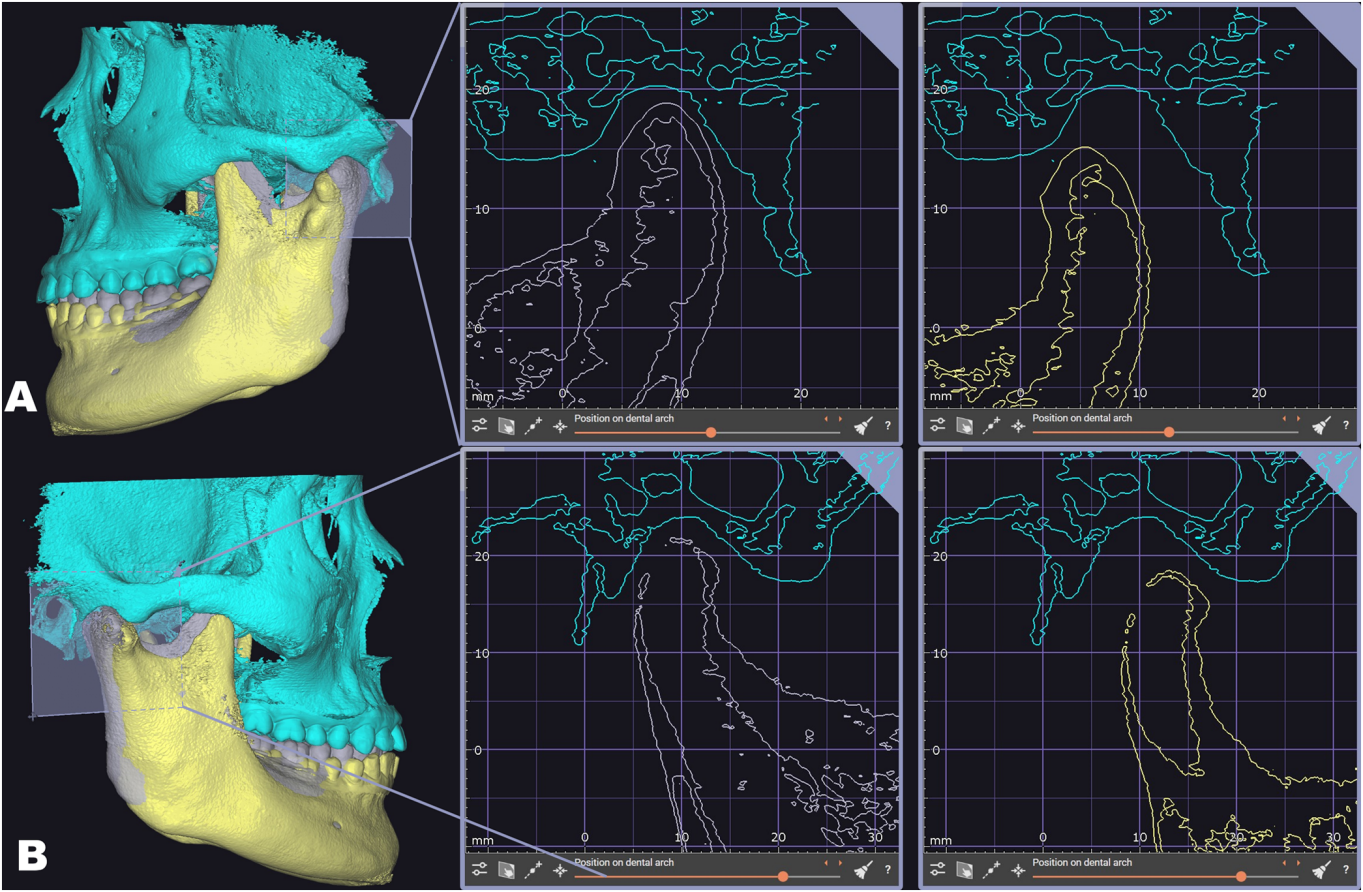
Determination of the anterior position of the mandibula though VDP. The gray mandible represents the initial position, while the yellow mandible indicates the established forward position. **(A)** The left positional relationship between the condyle and the fossa when the mandible was in its initial and anterior positions; **(B)** the right positional relationship between the condyle and the fossa when the mandible was in its initial and anterior positions.

Through VDP, we began the design process for personalized functional anterior repositioning splint (FARS). Our initial goal was to identify the anterior position of the mandibula. Based on the principles of ARS therapy, we aimed to shift the condyle forward and downward, thereby augmenting the interspace between the condyle and the skull during the forward movement of the mandibula. This modification facilitates the repositioning of the anterior disc to its normal location. Utilizing recordings of mandibular movements, we gradually protruded the mandible of the VDP until the condyles surpassed the stagnation point, indicating that the disc has return to normative positions (Figure 2).

With VDP, the opening degree between maxilla and mandibula was manageable in order to obtain sufficient space to accommodate FARS, the position relationship between condyle and skull was visible in order to verify the accuracy of the anterior position, the height between tooth of maxilla and mandibula was measurable in order to design the occlusal surface morphology and the protruded distance was controllable in order to reduce discomfort for the patient.

**Figure 3:**
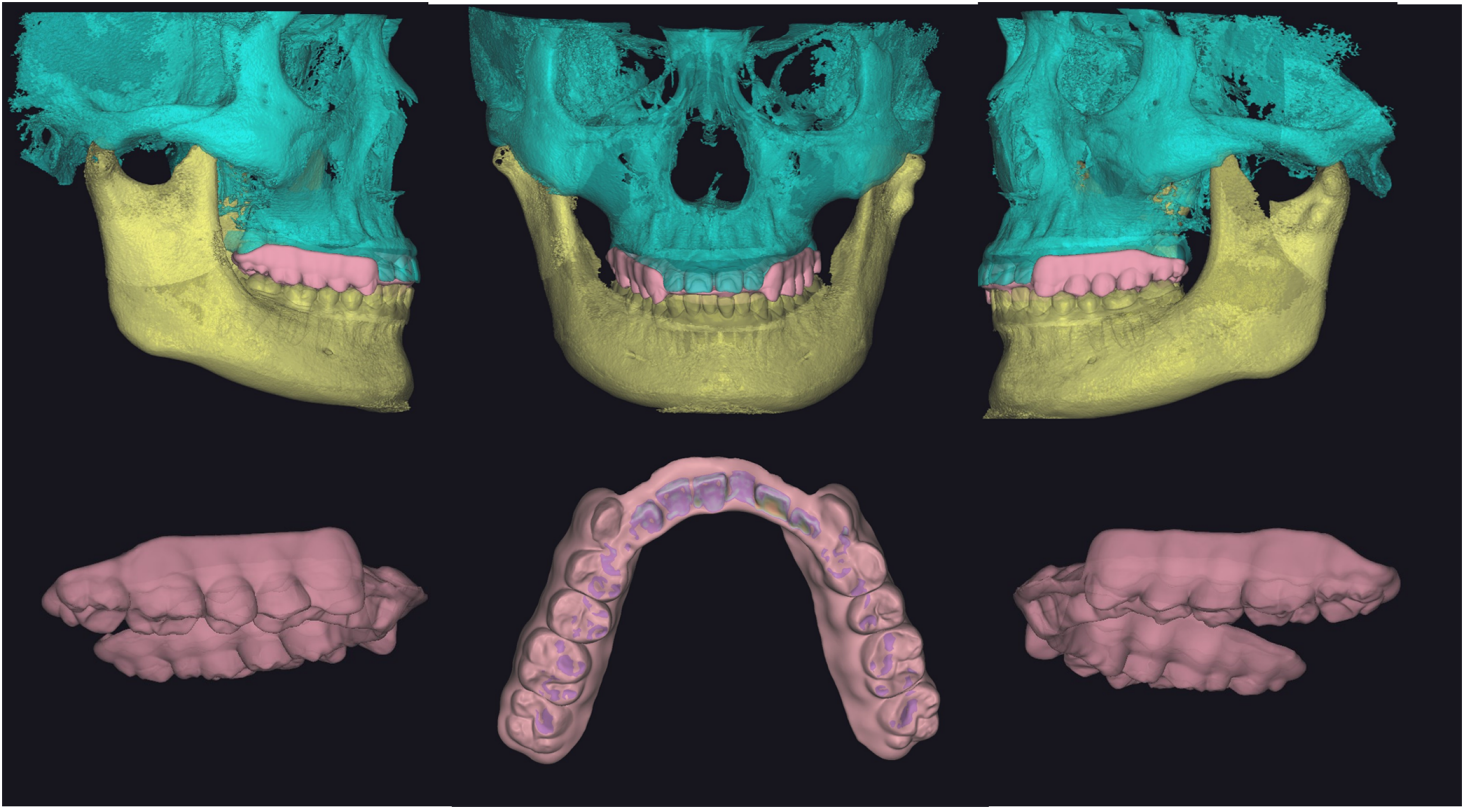
The morphology of the occlusal surface of the FARS we designed in accordance with the patient's specific dynamic occlusion. Additionally, there is a guard located on the palatal side of the front teeth area that prevents the retraction of the lower jaw.

Once we established this position, we designed the FARS with a guard to prevent the retraction of the anterior mandibula. Additionally, we tailored the morphology of the occlusal surface of the FARS to facilitate stable contact and lateral guidance between the occlusal plate and the opposing dentition, according to the patient's specific dynamic occlusion. The FARS we developed was intended for use on the patient's maxillary arch, with a recommended thickness of approximately 2 mm (Figure 3).

Finally, we employed 3D printing technology to fabricate FARS. (Chairside Pro 3D Printer, China). The material we used was Surgical Guide UV, which was odorless, biocompatible, easy to polish, possessed high mechanical strength, had low water absorption, and wass suitable for prolonged intraoral use.

#### 2.2.3 Clinical evaluation and patient satisfaction

**Figure 4:**
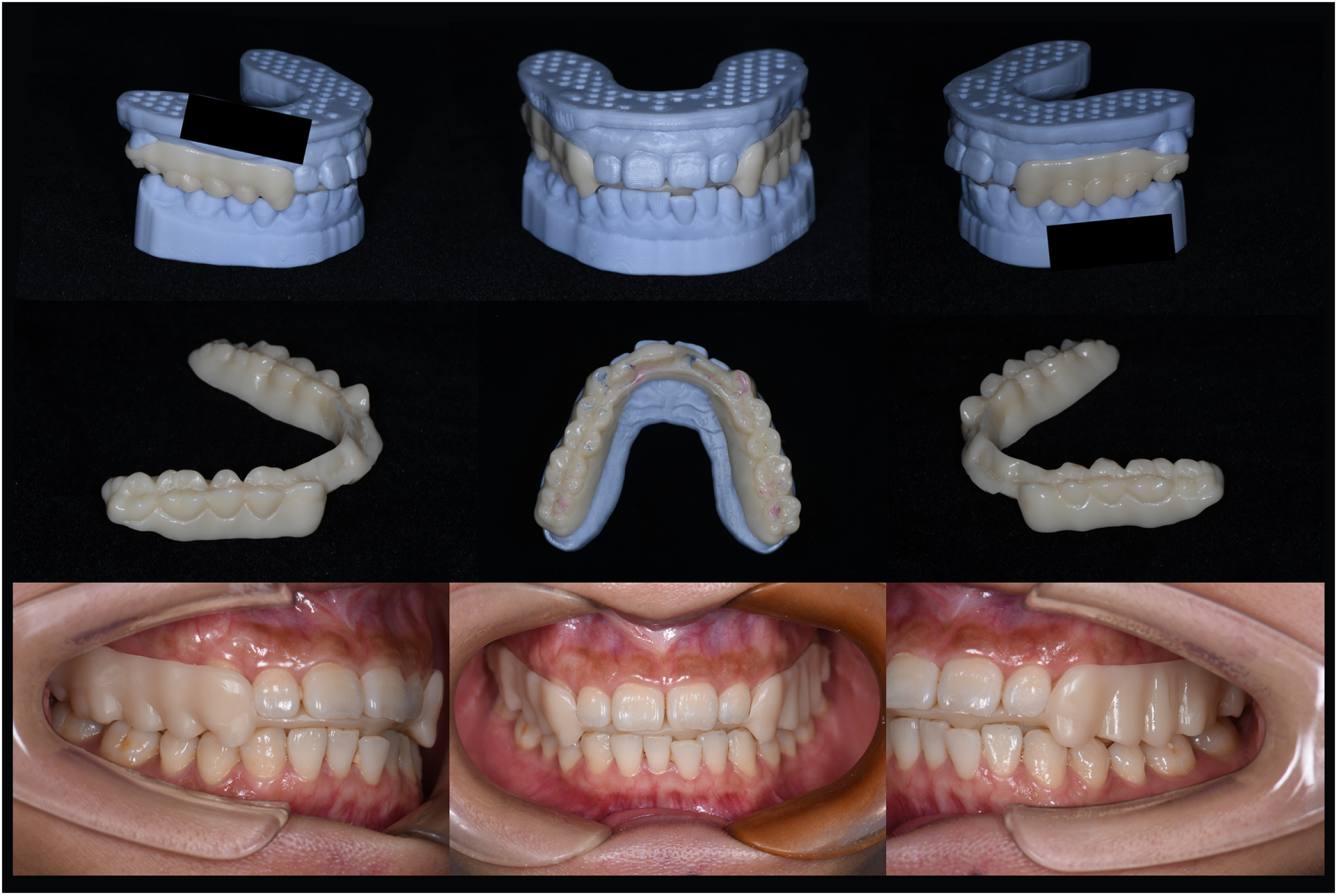
There was no interference with masticatory function during the oral movement tests after the patient wore the FARS.

The patient was required to wear FARS and perform various oral movements. Because we comprehensively considered the anterior extension guidance function of FARS and the appropriate mouth opening of the patient, the patient's mandibula could be guided and fixed stably after wearing the FARS. Consequently, the patient was able to consume food comfortably without any adverse effects (Figure 4).

### 2.3 Follow-up and outcomes

The patient was instructed to wear the FARS throughout the day. Following a three-month period of therapy, the patient reported a great reduction in jaw pain and clicking noise. She exhibited satisfactory masticatory function without any discomfort. The condition of the TMJ improved, and the mandibular position stabilized (Supplementary Figure S3).

The patient is presently undergoing orthodontic treatment, during which the orthodontist has adjusted the occlusal relationship between the upper and lower teeth, ensuring an appropriate position relationship between the condyle and the skull. We will continue to monitor the patient's treatment outcomes.

## 3 Discussion

The principle of the ARS therapy to ADDwR is predicated on the alteration of the position relationship of the patient's TMJ following the application of the ARS. This intervention facilitates the downward and forward movement of the condyle, enabling it to “catch up” with the anterior disc, and recombine with it to restore a stable disc-condyle complex. Research has indicated that patients who wear ARS over extended periods exhibit favorable outcomes in terms of disc restoration (9).

However, due to the lack of standardized operating instructions or authoritative explanations of the mechanisms of the therapy, doctors often feel trapped when applying this therapy because they are unable to directly observe whether the patient's articular disc returns to the correct position after wearing the ARS. Besides, traditional ARS designs predominantly prioritize functionality while overlooking the potential adverse effects of the thick ARS on masticatory function, which may lead to patient discomfort.

Conventionally, the anterior position is employed as an indirect measure to ascertain whether the condyle has successfully repositioned itself in relation to the disc. Patients are typically instructed to open their mouths gradually a clicking sound is perceived, which is referred to as the “anterior position.” According to Chen's research (10), 93.5% of patients had an ideal disc-condyle relationship in the edge-to-edge position. However, some other scholars have different opinions. Research by Liu (11) showed that the anterior position of joint clicking was usually less than the protruded position with the incisors in an edge-to-edge relation, and the effect of ARS might be influenced if the forward extension distance was insufficient. Some researchers argued that excessive mandibular protrusion can hinder patients' ability to eat or speak, thereby contributing to an unstable masticatory system, which may serve as a potential catalyst for TMDs (12).

In subsequent regular follow-ups, improvements in the patient's TMJ condition have been observed, with normal and stable mandibular movement reported. With the guidance for subsequent treatment provided by this study, the accuracy of treatment can be improved, and the cumbersome from repeated confirmation and adjustment during treatment can be reduced. Patients can quickly adapt and exercise chewing function normally after wearing the FARS. Additionally, advancements in digital manufacturing technology may enhance processing efficiency and product performance. However, ongoing monitoring is essential to determine whether the patient's disc may revert to an anterior position.

This study implemented an advanced fully digital diagnosis and treatment process, which continues to encounter several challenges in practical applications. These challenges are primarily associated with the financial investment required for equipment procurement and the learning curve faced by clinical practitioners. Advancements in technology have led to the development of contemporary digital products that emphasize integration and multifunctionality, aiming to achieve comprehensive clinical functional integration within a single device. The intraoral scanner and mandibular movement recording device utilized in this study exemplify this trend, as their clinical functionalities are continually expanding and being optimized. From an industry development perspective, as technology matures and the market expands, it is anticipated that the economic costs associated with equipment and software will gradually decrease. Concurrently, the implementation of standardized and intelligent operating systems is expected to significantly alleviate the learning challenges faced by clinical practitioners, thereby enhancing the efficiency of technology dissemination.

Finally, this study has several limitations: first, the assessment of efficacy largely depends on subjective patient feedback and MRI results, which may introduce bias. Second, as an initial investigation into FARS treatment methods, the study's sample size is small, and the generalizability of the findings requires further validation. To thoroughly assess the effectiveness of FARS treatment, more extensive research is necessary, including large-scale clinical trials to confirm the reliability and reproducibility of the FARS treatment process; the introduction of quantitative evaluation metrics, such as objective measures like chewing function tests and muscle function tests conducted before and after treatment; the design of rigorous controlled clinical trials to systematically compare the efficacy of FARS with traditional treatment methods; and the establishment of a large sample cohort for long-term follow-up to evaluate the long-term efficacy and safety of FARS treatment.

## 4 Conclusion

In this case, we improved the traditional ARS therapy with the establishment of a novel digital workflow to fabricate FARS based on the virtual dental patient for ADDwR. The predictability and visualization of treatment effect were ensured by the controllable and visualized virtual dental patient based on multimodal data fusion with reliable target jaw positions and accurate movement recording. Furthermore, the masticatory function of the patient was preserved through the design of the occlusal surface morphology. This approach represents a significant optimization and advancement in the management of ADDwR. However, it is important to note that this personalized FARS therapy is time-intensive, and further investigation is required to assess the long-term efficacy of the therapy.

## Data Availability

The original contributions presented in the study are included in the article/Supplementary Material, further inquiries can be directed to the corresponding author.
