# Supplementary material for: A novel digital workflow to fabricate anterior repositioning splint with fully masticatory function based on virtual dental patient for anterior disc displacement with reduction: a case report
Source: Front Oral Health. 2025 Mar 12;6:1534571. doi: 10.3389/froh.2025.1534571 (PMC11936970; doi:10.3389/froh.2025.1534571)
Supplement: Supplementary file 1 [file Datasheet1.docx]

Supplementary Material

A novel digital workflow to fabricate anterior repositioning splint with fully masticatory function based on virtual dental patient for anterior disc displacement with reduction: a case report

**Weicai Liu^1*^†, Xinhuan Zhao^1^†, Jiefei Shen^2^, Ran Wei^3^**

1 Department of Prosthodontics, School & Hospital of Stomatology, Tongji University, Shanghai Engineering Research Center of Tooth Restoration and Regeneration, Shanghai, China

2 State Key Laboratory of Oral Diseases, National Clinical Research Center for Oral Diseases, Department of Prosthodontics, West China Hospital of Stomatology, Sichuan University, Chengdu, Sichuan, China.

*** Correspondence:** Weicai Liu and Xinhuan Zhao are co-first authors of the article. They contributed equally to this work.

**Corresponding Author:** Weicai Liu

vogi@163.com; weicai_liu@tongji.edu.cn

**Supplementary Data**


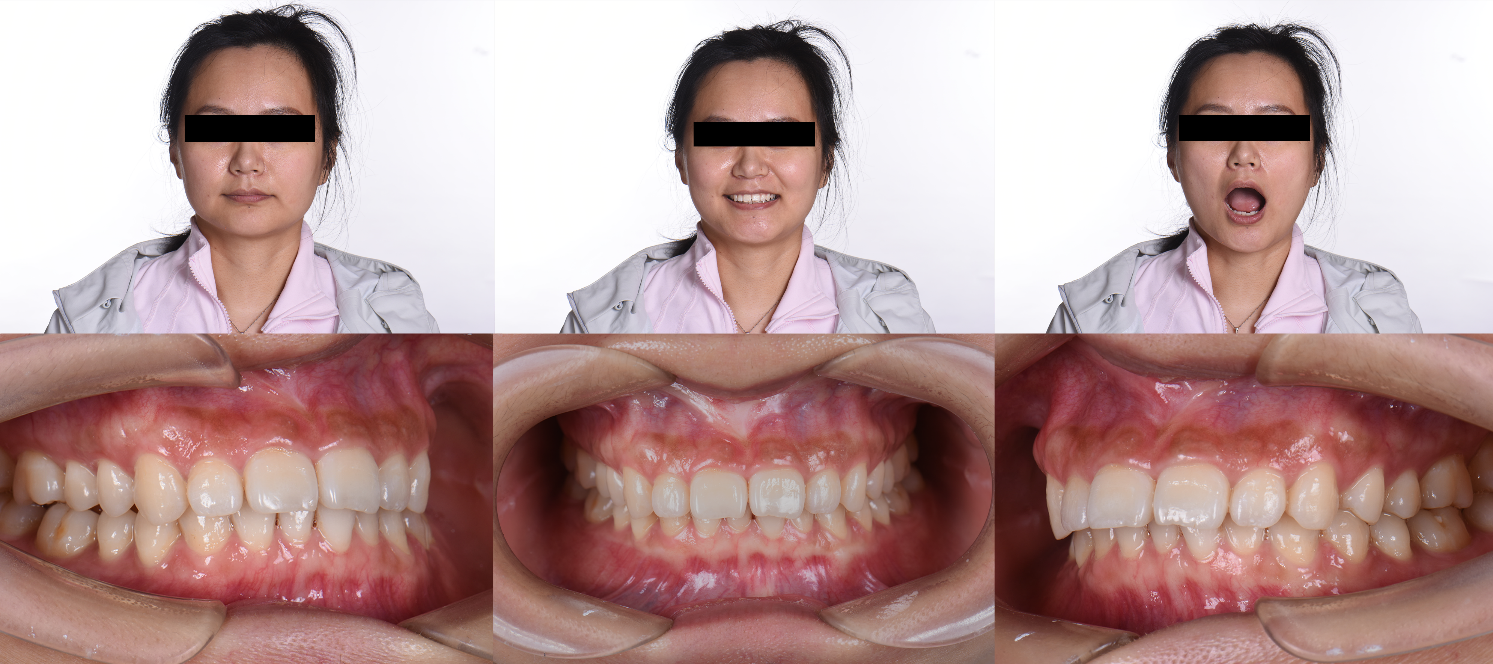


**Supplementary Figure 1.** Pretreatment facial photographs and intraoral photographs of the patient.


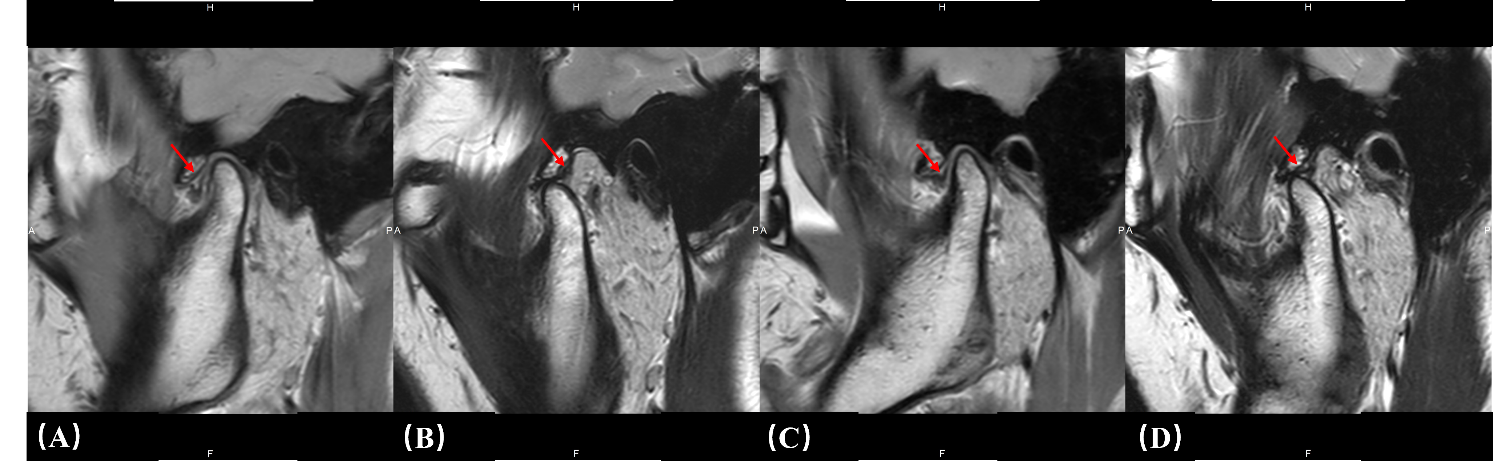


**Supplementary Figure 2.** MRI images of the patient before treatment. A. When the maxilla and mandible were in the intercuspal position (ICP), the left temporomandibular joint disc exhibited a state of anterior displacement. B. When the maxilla and mandible were in the maximum opening position, the left temporomandibular joint disc returned to the glenoid fossa. C. When the maxilla and mandible were in the intercuspal position (ICP), the right temporomandibular joint disc exhibited a state of anterior displacement. D. When the maxilla and mandible were in the maximum opening position, the right temporomandibular joint disc returned to the glenoid fossa.


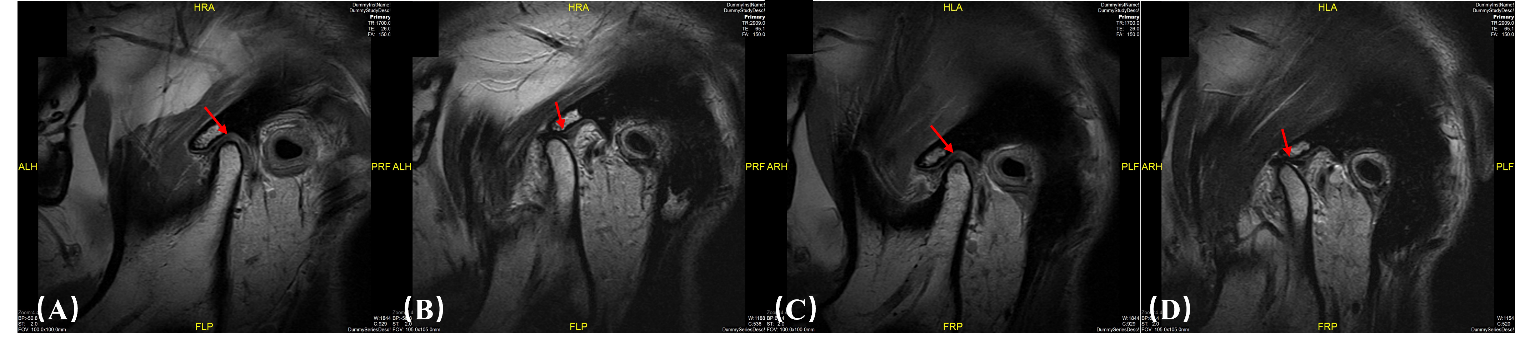


**Supplementary Figure 3.** MRI images of the patient before treatment. In comparison to the MRI images presented in supplementary figure 2, the bilateral temporomandibular joint discs consistently remained positioned within their respective glenoid fossae, regardless of whether the maxilla and mandible were in the intercuspal position or the maximum opening position.
